# Impotency

**Published:** 1882-10

**Authors:** 


### IMPOTENCY.

In the American Medical Digest Dr. Richardson makes the statement that "celerina" is the most efficient nerve tonic in the materia medica. *To make the matter sure he adds ten grains of bromide* to each teaspoonful dose. This precaution appears to be a very wise one indeed. He states that the patient after commencing the use of this formula rarely if ever has an emission. This clearly proves that "celerina" is a good nerve tonic and—that it will stop the emission if *bromide be added to it.—Chicago Med. Review.*

